# Supplementary material for: A Phase II Study of Glembatumumab Vedotin for Metastatic Uveal Melanoma
Source: Cancers (Basel). 2020 Aug 13;12(8):2270. doi: 10.3390/cancers12082270 (PMC7465139; doi:10.3390/cancers12082270)
Supplement: Supplementary file 1 [file cancers-12-02270-s001.pdf]

Supplementary Material: A Phase II Study of Glembatumumab Vedotin for Metastatic Uveal Melanoma

Supplementary Table 1: Pre and Post Treatment GPNMB Expression				
Tissue Site	Baseline % Positive	Intensity	Day 21 % Positive	Intensity
Liver	100	3+	100	3+
Liver	100	3+	100	3+
Liver	50	1+	-	-
Breast	0	0	-	-
Liver	0	0	0	0
Liver	0	0	20	1+
Skull base, subcutaneous	100	3+	100	3+
Liver	100	3+	100	3+
Liver	20	1+	30	1+
Liver	0	0	-	-
Liver	-	-	0	0
Liver	70	3+	0	0
Liver, Subcutaneous	100	2+	100	3+
Eye (primary), Lymph node	80	3+	100	3+
Liver	100	3+	100	3+
Liver	100	2+	99	3+
Liver	-	-	15	3+
Liver	100	3+	80	3+
Subcutaneous	100	3+	100	3+
Subcutaneous	70	3+	100	3+
Subcutaneous	35	2+	100	3+
Eye (primary), Subcutaneous	100	3+	100	3+
Liver	95	3+	100	3+
Liver	0	0	45	2+
Liver	0	0	0	0
Liver	0	0	20	1+
Liver	30	2+	100	3+
Liver	100	3+	-	-

Abbreviations: GPNMB: Glycoprotein NMB

GPNMB expression was analyzed via IHC at baseline and after 1 cycle of GV. A change in GPNMB expression greater than 10% was considered a positive signal.
